# Pulmonary Aspergillosis Complicated by Hemophagocytic Lymphohistiocytosis: A Case Report and Literature Review

**DOI:** 10.7759/cureus.30908

**Published:** 2022-10-31

**Authors:** Rafal S Ali, Mitali Sen, Irene J Tan

**Affiliations:** 1 Internal Medicine, Einstein Medical Center Montgomery, East Norriton, USA; 2 Rheumatology, Einstein Medical Center Philadelphia, Philadelphia, USA

**Keywords:** neurological signs and symptoms, secondary hlh, immune-mediated inflammatory disorder, pulmonary aspergillosis, hemophagocytic lymphohistiocytosis (hlh)

## Abstract

Hemophagocytic lymphohistiocytosis (HLH) is a life-threatening syndrome involving excessive immune activation. It can be primary (familial) or secondary (triggered by infection, malignancy, or rheumatological disease).

This is a case of a previously healthy 43-year-old African American woman who presented with fever and confusion. The patient was eventually diagnosed with pulmonary aspergillosis and responded well to antifungal therapy. She met the diagnostic criteria of HLH-2004 trial for hemophagocytic lymphohistiocytosis. She also fulfilled the 2019 classification criteria for systemic lupus erythematosus (SLE) without the classical signs and symptoms of SLE.

HLH management includes supportive management, treatment of underlying condition, and immunosuppressive treatment. Etoposide and dexamethasone are commonly used treatments for HLH; however, underlying active infection can limit the treatment options. In our case, the patient was treated with steroids and hydroxychloroquine. Her condition gradually improved and she recovered without complications.

Based on our literature review, we encountered six cases of HLH secondary to Aspergillosis with a mean age of approximately 47 years. The diagnosis of HLH is often delayed because of nonspecific presentation. Early identification and treatment are crucial to improve the survival rate.

## Introduction

Hemophagocytic lymphohistiocytosis (HLH) is a life-threatening syndrome of inflammation and tissue injury secondary to excessive activation of the immune system. It has a wide range of nonspecific symptoms that make the diagnosis difficult. Familial and sporadic cases were reported. The disease can affect any age, but it is more common in the pediatric population.

The pathogenesis is believed to be secondary to the failure of normal elimination of activated macrophages by cytotoxic lymphocytes and natural killer cells leading to poor macrophage function, production of large amounts of cytokines, and phagocytosis of host cells which cause excessive inflammation and tissue injury. Cytokines can be found elevated in HLH, including interferon gamma, chemokine CXCL9, tumor necrosis factor alpha (TNF alpha), interleukin-6, interleukin-10, interleukin-12, and soluble interleukin-2 receptor (CD25) [1-4].

HLH can be triggered by infections, malignancies, and rheumatological disorders. Infectious triggers include viruses (Epstein-Barr virus (EBV), cytomegalovirus (CMV), parvovirus, herpes simplex virus, varicella-zoster virus, measles virus, human herpes virus 8, H1N1 influenza virus, and human immunodeficiency virus (HIV)), bacteria, parasites and fungi. Mutations in genes that cause familial hemophagocytic lymphohistiocytosis (FHL) are commonly found in younger patients. Several genes have been detected in familial cases, including STX11, PRF1, and UNC13D. The disease may be isolated or recurrent. Recurrent HLH usually occurs with a genetic predisposition [5].

## Case presentation

We present a case of a 43-year-old African-American female with no significant past medical or surgical history who was brought to the hospital after being found confused.

Vital signs were significant for a temperature of 39.1°C. The physical examination revealed bilateral basal and mid-lung crackles. Initial laboratory tests showed low neutrophil, red blood cell, and platelet counts. Hemoglobin was low and serum creatinine was elevated. Ferritin was significantly increased. She also had elevated aspartate aminotransferase (Table 1).

**Table 1:** laboratory tests Neut: neutrophils, Hb: hemoglobin, RBC: red blood cell, Plt: platelet, S.creatinine: serum creatinine, ALT: alanine transaminase, AST: aspartate aminotransferase, TGL: triglycerides, LDH: lactate dehydrogenase, ANA: antinuclear antibody, ab: antibodies, anti-RNP: antinuclear ribonucleoprotein

Laboratory tests	On the day of presentation	Two days after presentation	Reference range
Neut	1,000/µl	1,300/µl	2500-8000/µl
Hb	8 g/dl	8.5 g/dl	11.6-15 g/dl
RBC	2.82 million/ µl	2.79 million/ µl	3.92-5.13 million/µl
Plt	34,000/ µl	31,000/ µl	150.000-450.000/µl
S. Creatinine	1.33 mg/dl	0.94 mg/dl	24-336 ng/ml
Ferritin	5,838 µg/l	6,432 µg/l	0.59-1.04 mg/dl
ALT	40 IU/l	32 IU/l	19-25 IU/L
AST	250 IU/l	160 IU/l	10-40 IU/L
Additional laboratory tests two days after presentation			
TGL	221 mg/dl		<150 mg/dl
LDH	872 IU/l		<140 IU/l
ANA	1:80		≤1:40
D-Dimer	>2500 ng/ml		220-500 ng/ml
Anti-RNP ab	Positive		Lab did not specify
Anti-Smith ab	8 U/ml		0-7 U/ml
C3	80 mg/dl		88-201 mg/dl

**Figure 1:**
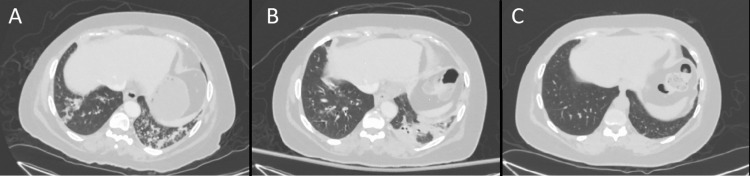
CT chest CT: computerized tomography scan; A: CT chest upon presentation showing bilateral pneumonia with the largest consolidation in the left lower lobe, B: follow-up CT chest a few days after presentation showing persistence of bilateral pneumonia, C: follow-up CT chest two months after presentation showing resolution of bilateral pneumonia.

A computerized tomography (CT) scan of the chest, abdomen and pelvis was performed and showed evidence of bilateral pneumonia (Figure 1A). The patient also underwent brain imaging (including CT scan, magnetic resonance imaging (MRI), and magnetic resonance angiogram (MRA)) to evaluate her confusion, and these tests were unremarkable for any acute changes.

Blood and urine cultures were taken, and she was started empirically on intravenous broad-spectrum antibiotics (cefepime and azithromycin). Sputum culture showed normal respiratory flora. Blood and urine cultures remained negative, but she continued to be febrile, and a repeated CT scan of the chest five days later showed the persistence of bilateral pneumonia (Figure 1B).

Two days later, laboratory tests showed consistently low WBC, RBC and platelet counts. She also had elevated triglycerides, lactate dehydrogenase (LDH), and D-dimer levels. Antinuclear antibody (ANA) came back positive and C3 level was low. Antinuclear ribonucleoprotein (anti-RNP) and anti-Smith antibodies were positive (Table 1).

Given the lack of improvement on empiric antibiotics, the decision was made for bronchoscopy, and the bronchoalveolar lavage culture was positive for *Aspergillus fumigatus*. The patient was started on intravenous micafungin and her fever resolved, micafungin was switched afterward to oral voriconazole (plan to complete six months of therapy).

She fulfilled the 2019 classification criteria for systemic lupus erythematosus (SLE) (positive ANA of 1:80, with additive criteria: fever, leukopenia, thrombocytopenia, positive anti-Smith antibody, low C3, and confusion/delirium), so she was started on hydroxychloroquine (400 mg daily).

Hemophagocytic lymphohistiocytosis (HLH) was suspected because of the significant elevation of ferritin, pancytopenia, fever, and elevated triglyceride levels. The H-score for HLH was calculated and showed a 45% probability of HLH. She fulfilled four out of eight criteria of the HLH-2004 trial, which was not sufficient to diagnose HLH. Bone marrow biopsy and soluble IL-2 receptor alpha (CD25) testing were recommended. CD25 level was elevated at 2,577 (532-1891 pg/ml). Bone marrow biopsy was unsuccessful. With an elevated CD25 level, the patient fulfilled five out of eight criteria of the HLH-2004 trial diagnostic criteria and she was diagnosed with hemophagocytic lymphohistiocytosis secondary pulmonary aspergillosis. She was started on intravenous methylprednisolone which was switched later to oral prednisone. For leukopenia, the hematologist started Granix® (granulocyte colony-stimulating factor - Teva Pharmaceutical Industries Ltd., North Wales, USA) and her leukopenia resolved.

Extensive infectious work-up (for CMV, urine Legionella antigen, COVID-19 test, respiratory panel, viral hepatitis panel, and EBV) was unremarkable. The patient was followed by multidisciplinary medical teams (rheumatology, hematology, infectious disease and pulmonology teams). She continued to improve gradually and was discharged after a month of hospital stay.

On a follow-up visit (one month after discharge) with rheumatology and infectious disease specialists, pancytopenia was resolved, inflammatory markers normalized, and C3 level was normal. She had no recurrence of her neurological or respiratory symptoms. Two-month follow-up thoracic CT scan showed a resolution of bilateral pneumonia (Figure 1C). The patient was sent for genetic testing and natural killer cell activity test; unfortunately, she did not have the tests done and did not follow up afterward.

## Discussion

We describe the case of a previously healthy woman who developed HLH secondary to pulmonary aspergillosis. HLH can develop secondary to infections, including fungal infections. The patient did not have a known environmental exposure to aspergillosis. She also did not have a known immunodeficiency status; however, the possible underlying autoimmune rheumatological disease can be a precipitating factor to develop the invasive infection.

Neurological symptoms can be the predominant manifestation of HLH, including changes in mental status, seizures, nerve palsies, focal weakness, and encephalitis, with up to one-third of HLH cases presenting with neurological manifestations [6]. Our case presented with confusion. Pulmonary involvement has been reported in up to 40% of patients, and can include acute respiratory distress syndrome (ARDS) or infections. Our patient showed evidence of bilateral pneumonia upon presentation [7].

Diagnosis of HLH can be determined by HLH-2004 trial criteria, which include either confirmation of HLH-associated genetic mutation or the presence of five of eight diagnostic criteria (Table 2) [8]. While evidence of hemophagocytosis can support HLH, it is not required to make the final diagnosis.

**Table 2:** Diagnostic criteria of HLH-2004 trial [8] HLH: hemophagocytic lymphohistiocytosis

Molecular identification of an HLH-associated gene mutation:	Or	5 of 8 of the following criteria:
Children require documentation of homozygosity or compound heterozygosity for HLH-associated gene mutations		1. Fever
		2. Splenomegaly
		3. Cytopenia affecting 2 lines
Adults: heterozygosity may be sufficient if they have clinical findings associated with HLH		a. Hemoglobin <9 g/dl
		b. Platelets <100 k/μl
		c. Neutrophils <1.0 x10^9^/l
		4.Hypertriglyceridemia and/or hypofibrinogenemia
		a. Triglycerides > 265 mg/dl
		b. Fibrinogen<150 mg/dl
		5. Hemophagocytosis in bone marrow, spleen, liver or lymph nodes
		6. Low or absent natural killer cell activity
		7. Ferritin > 500 ng/ml
		8. CD25 >2400 U/mL

In our case, the diagnosis was made after fulfilling five of the eight HLH-2004 trial criteria, including fever ≥38.5°C, pancytopenia, elevated triglyceride level, highly elevated ferritin level, and elevated soluble CD25 (soluble interleukin-2 receptor alpha) level.

The patient also tested positive for ANA, anti-Smith, and anti-RNP antibodies, with no classical signs and symptoms of connective tissue disease. She fulfilled the 2019 classification criteria for SLE. SLE is a known risk factor for HLH, and the possible underlying rheumatological disease could have predisposed the patient to excessive immune activation in response to invasive infection.

High fever with markedly elevated ferritin can raise suspicion for adult-onset Still's disease; however, the absence of rash and joint involvement makes the diagnosis unlikely in our case.

HLH treatment includes supportive therapy and treatment of the underlying triggering conditions (infection, malignancy, or rheumatological disease). In severe cases, treatment with immunosuppressive drugs is indicated, these include dexamethasone, etoposide, intravenous immunoglobulin (IVIG) therapy, anakinra (interleukine-1 receptor antagonist) and other immunosuppressive medications. Given the invasive pulmonary aspergillosis in our case, the decision was made to treat her with IV steroids together with hydroxychloroquine, which helped to improve her condition.

Based on our literature review, we encountered six cases of HLH secondary to aspergillosis with a mean age of ~47 years (Table 3) [8-13].

**Table 3:** Cases of HLH secondary to Aspergillus infection BAL: brohchoalveolar lavage, BM: bone marrow, HLH: hemophagocytic lymphohistiocytosis, NK: natural killer cells, ANA: antinuclear antibodies,

Case	Age In years	Presentation	ferritin	BM biopsy	BAL	Other significant laboratory tests	Diagnosis	Treatment	Outcome
Karakosta et al., 2021 [8]	68	Fever and respirator distress	4062 ng/ml then increased to 23,863 ng/ml	histiocytosis	Revealed Aspergillus flavus	Elevated Triglycerides and pancytopenia	Acquired Hemophagocytic lymphohistiocytosis secondary to invasive pulmonary aspergillosis and Turicella Otitidis bacteremia	Broad spectrum antibiotics, Itraconazole and IV Hydrocortisone	death
Schouten MJG et al., 2017 [9]	38	Respiratory failure and fever	2961 ng/ml	Not done	positive for influenza-A and aspergillus fumigatus	Low NK activity	HLH secondary to influenza and invasive pulmonary aspergillosis	-Anakinra (interleukin-1 receptor antagonist), Antiviral (oseltamivir), Antifungal (amphotericin-B and voriconazole) and Interferon-gamma	death
Umekawa et al., 2020 [10]	54	Fever, dyspnea and skin rash	14.790 ng/ml	not done	Revealed Aspergillus fumigatus	ANA 1:80, elevated CD25 3,970 pg/ml and Decreased NK cell activity	Secondary Hemophagocytic Lymphohistiocytosis Associated with Invasive pulmonary Aspergillosis and the patient was eventually diagnosed with dermatomyositis	IVIG and antifungal therapy	Improved then the patient developed recurrence of secondary HLH associated with disseminated tuberculosis
Paul M, et al., 2022 [11]	68	Fever, Acute respiratory distress and Impaired consciousness	141,918 ng/ml	hemophagocytosis	BAL was not done, the patient had positive serum galactomannan	Elevated triglycerides	HLH secondary to mucormycosis and aspergillosis coinfection	Etoposide, steroid therapy, Tocilizumab and Antifungal therapy	death
Gandotra A, et al., 2022 [12]	47	Fever and respiratory distress in a liver transplant recipient who was recovering from severe COVID-19 infection	27,717 ng/ml	hemophagocytosis	Not done	Sputum culture was positive for acid fast bacilli and sputum fungal culture was positive for Aspergillus Flavus	Invasive pulmonary Aspergillosis and Tuberculosis complicated by HLH in sequelae of COVID-19 in a liver transplant recipient	IVIG, Antitubercular therapy and Antifungal therapy	recovered
Bello, A et al., 2018 [13]	9	Fever with respiratory distress	14,000 ng/ml	Not done	Not done	Elevated triglycerides	Aspergillus induced HLH in child with acute lymphoblastic leukemia	Antifungal therapy	recovered

On her follow-up appointment after discharge, she was sent for HLH genetic analysis which tests for many genes involved in the pathogenesis of primary HLH, she did not have the test done (possibly due to the cost), and she did not follow up afterward. Genetic testing can help to determine the risk of HLH recurrence, and the future need for hematopoietic cell transplant. If the patient has a genetic predisposition for HLH, this indicates testing of other family members for these genes, keeping in mind that the chance of finding a gene mutation is higher in the younger patients. In a study of 175 adults (age range, 18 to 75 years), only 14 percent had gene mutations [14]. Gene defects include mutations at FHL loci which code for cytotoxic granule formation and release pathway (PRF1/Perforin, UNC13D/Munc13-4, etc.), gene defects also include mutations that cause congenital immunodeficiency syndromes which are associated with an increased risk of HLH (Griscelli syndrome/RAB27A mutation, Chediak-Higashi syndrome/CHS1/LYST mutation, etc.) [15-18].

## Conclusions

HLH is a syndrome of intense immune activation and is a life-threatening condition that usually presents with non-specific symptoms. Without a high index of suspicion, diagnosis may be delayed, which can increase the mortality rate. Testing for fungal infection is an important part of secondary HLH workup. We also recommend checking the ferritin level whenever HLH is suspected since it is considered as a hallmark of HLH and has been considered in the literature as prognostic biomarker for high mortality. Finally, bone marrow biopsy can help to confirm HLH but is not necessary for the diagnosis.
